# How Simian Virus 40 Hijacks the Intracellular Protein Trafficking Pathway to Its Own Benefit … and Ours

**DOI:** 10.3389/fimmu.2018.01160

**Published:** 2018-05-28

**Authors:** Miguel G. Toscano, Peter de Haan

**Affiliations:** ^1^Amarna Therapeutics SL, Sevilla, Spain; ^2^Amarna Therapeutics BV, Leiden, Netherlands

**Keywords:** Simian Virus 40, polyomavirus, immune evasion, viral vector, non-immunogenicity, immune tolerance

## Abstract

Viruses efficiently transfer and express their genes in host cells and evolve to evade the host’s defense responses. These properties render them highly attractive for use as gene delivery vectors in vaccines, gene, and immunotherapies. Among the viruses used as gene delivery vectors, the macaque polyomavirus Simian Virus 40 (SV40) is unique in its capacity to evade intracellular antiviral defense responses upon cell entry. We here describe the unique way by which SV40 particles deliver their genomes in the nucleus of permissive cells and how they prevent presentation of viral antigens to the host’s immune system. The non-immunogenicity in its natural host is not only of benefit to the virus but also to us in developing effective SV40 vector-based treatments for today’s major human diseases.

## Introduction

As intracellular parasites, viruses hijack the host cell machinery to replicate, spread and survive. Host cells use membrane-bound and cytoplasmic receptors to sense pathogen-associated molecular patterns (PAMPs). After receptor-binding, viral structural proteins may serve as PAMPs and bind toll-like receptors (TLRs) that are located on the cell surface or on endosomal membranes. After replication, virus-specific RNAs serve as PAMPs and bind cytoplasmic RIG-I-like receptors (RLRs). Activation of TLRs or RLRs leads to the assembly of inflammasomes that induce an inflammatory response (1, 2). Inflammation is a highly orchestrated cascade of processes aimed at confining the infection and ultimately in inducing an adaptive immune response directed to peptides (antigens) derived from viral proteins that are presented on major histocompatibility (MHC) molecules on the surface of cells of the immune system.

Simian Virus 40 (SV40), the type member of the Polyomaviridae family, was discovered in the fifties of the previous century as a contaminating virus in the polio vaccines that in those days were produced in primary cells from macaques (3, 4). Since then, SV40’s DNA genome was the first animal virus genome to be characterized (5, 6). SV40 served as the model virus to study molecular and biochemical processes in eukaryote organisms (7). The first mammalian viral gene delivery vector was derived from SV40 (8) and pioneering gene transfer studies using replication-defective SV40 vectors ultimately resulted in the recent approval of the first viral vector-based gene therapies to the market (9), albeit that the currently used vectors are derived from adeno-associated virus (AAV) or the human immunodeficiency virus type 1 (HIV-1).

SV40 is a macaque polyomavirus consisting of icosahedral particles of 45 nm in diameter (10, 11). The virus particle consists of 72 pentamers of the major viral protein VP1. On the inside of the capsid, each pentamer forms a hydrophobic pocket that is bound to one monomer of the viral proteins VP2 or VP3 (12). Each particle contains a single copy of the viral genome, a circular 5.2 kilobase pairs long double-stranded DNA molecule packaged with histones to form a mini-chromosome. The SV40 genomic DNA has two genes. The early gene encodes two non-structural replication-associated proteins: small T antigen and large T antigen. The late gene codes for the structural viral proteins VP1, VP2, and VP3, respectively (13, 14).

**Figure 1:**
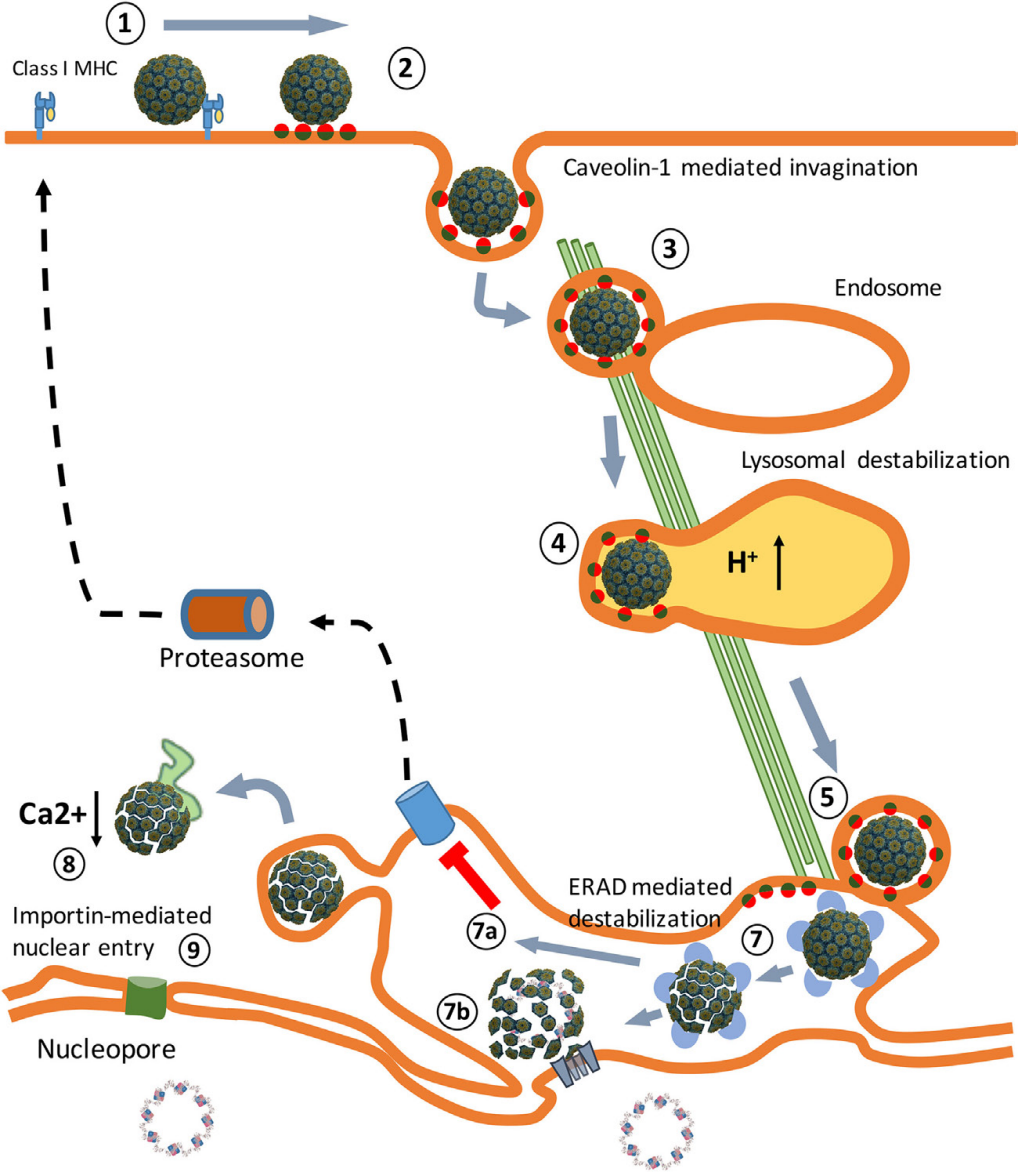
An overview of the Simian Virus 40 (SV40) cell entry process. (1) Binding of the virus particles to major histocompatibility class I (MHC-I) molecules that target them to lipid rafts, enriched in GM1 molecules. (2) Release from MHC-I molecules, binding to GM1 molecules, and caveolae vesicle formation. (3) Endosome internalization. (4) Endosome maturation and particle destabilization. (5) Release from endolysosomes and endoplasmic reticulum (ER) trafficking. (6) ER-associated degradation (ERAD)-mediated particle destabilization. (7a) ERAD-mediated cytosol transport. (7b) Viroporin-mediated nuclear entrance. (8) Cytosol destabilization. (9) Nucleopore-mediated nuclear entrance. The SV40 image was created using VMD software (19) PDB ID: 1SVA (20) and is a courtesy of Dr. J.-Y. Sgro, UW-Madison, USA (http://www.virology.wisc.edu/virusworld/).

In macaques, SV40 causes chronic asymptomatic infections (15). Children who received the SV40-contaminated poliovirus vaccine did not develop an adaptive immune response to the virus particles and excreted SV40 in their stools within 5 weeks after vaccination (16). This indicates that SV40 capsids do not serve as PAMPs and that the virus does not replicate in human cells. Studies in animals administered with replication-defective SV40 vector particles in the absence of adjuvants (PAMPs) do not result in the induction of an adaptive immune response to SV40, demonstrating that SV40 particles are non-immunogenic *in vivo* (17, 18). This implies that SV40 after its entrance into permissive cells (Figure 1) is able to efficiently evade TLR binding and prevents presentation of viral antigens on MHC molecules to cells of the host’s immune system.

The non-immunogenicity of SV40 combined with the absence of an immune memory for this macaque polyomavirus in the human population is of benefit to us, since it renders SV40 highly attractive for use as a gene delivery vector in gene and immunotherapies.

## Endocytosis

**Figure 2:**
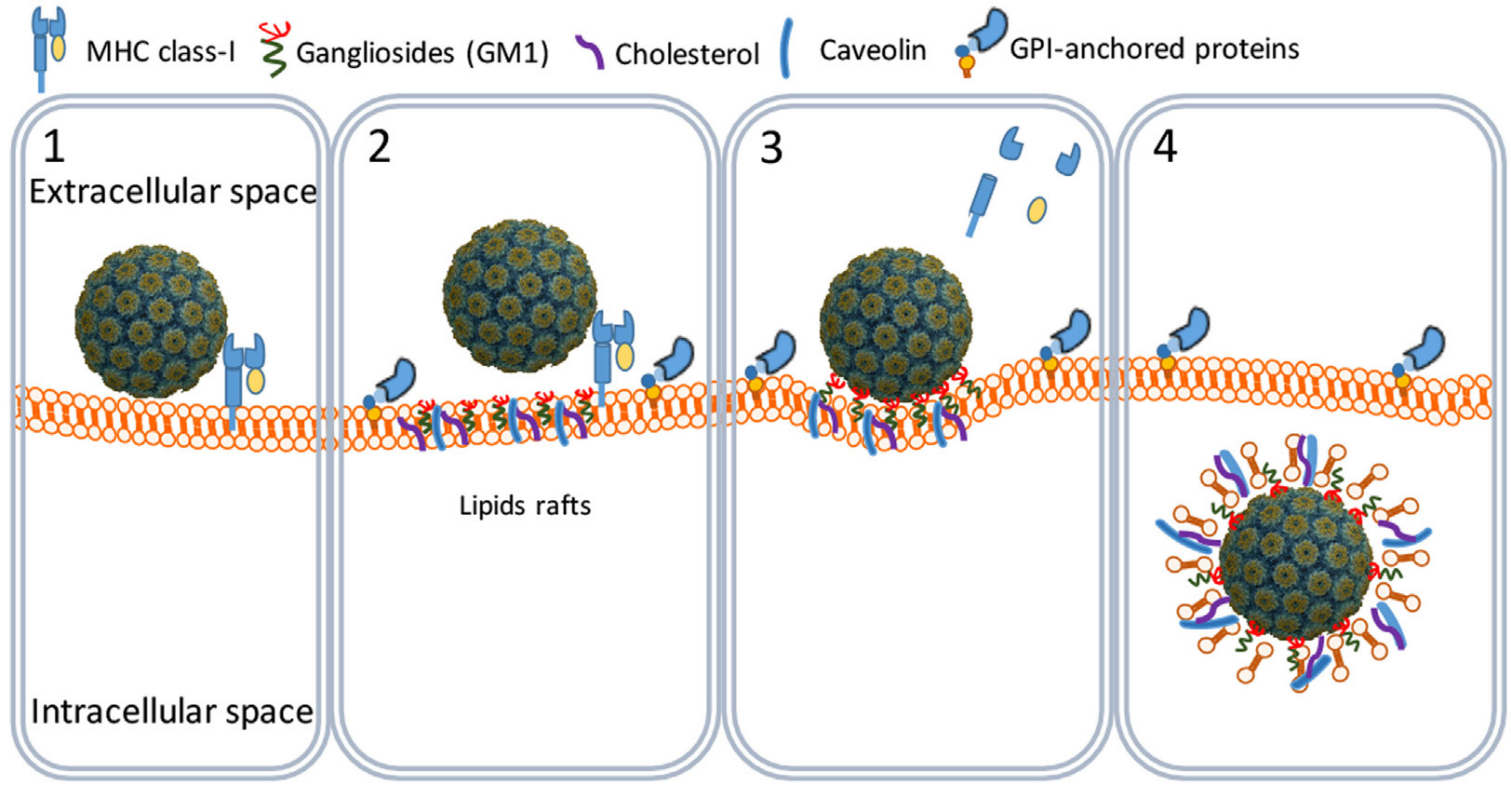
SV40 cell attachment and internalization. SV40 particles attach to the cell surface by binding to major histocompatibility class I molecules (1) that shuttle the virus particles to GM1-rich lipid rafts (2). The particles bind GM1 and induce the formation of caveolar endocytic vesicles (3) and get into the cytosol through an active translocation mediated by glycosylphosphatidylinositol (GPI)-anchored proteins (4).

SV40 binds MHC class I (MHC-I) molecules present on the surface of all body cells (21–23). Once bound, the SV40–MHC-I complexes migrate to caveolin-enriched membrane domains named caveolin pits also known as lipid rafts (Figure 2). Caveolin pits are cell surface membrane domains enriched in cholesterol, gangliosides, glycosphingolipids, and protein receptors including MHC-I molecules that are involved in endocytosis and transcytosis (24–27).

At the caveolin pits, the MHC-I molecules are degraded by metalloproteinases whereby the SV40 particles bind to membrane ganglioside GM1 molecules which are considered their endocytic receptors (28, 29). Binding of SV40 particles to GM1 induces a curvature of the cell membrane that results in the formation of endocytic vesicles known as caveolae (20, 24, 26, 30). Caveolae are circular or tubular vesicles of 70–100 nm in diameter and usually contain one SV40 particle. Next to GM1, cholesterol and tyrosine kinases are needed for the formation of caveolae, since nystatin (a cholesterol sequestering agent) and genistein (a tyrosine kinase inhibitor) efficiently block the translocation of SV40 particles into the caveolae (Figure 2) (29, 31–33).

**Figure 3:**
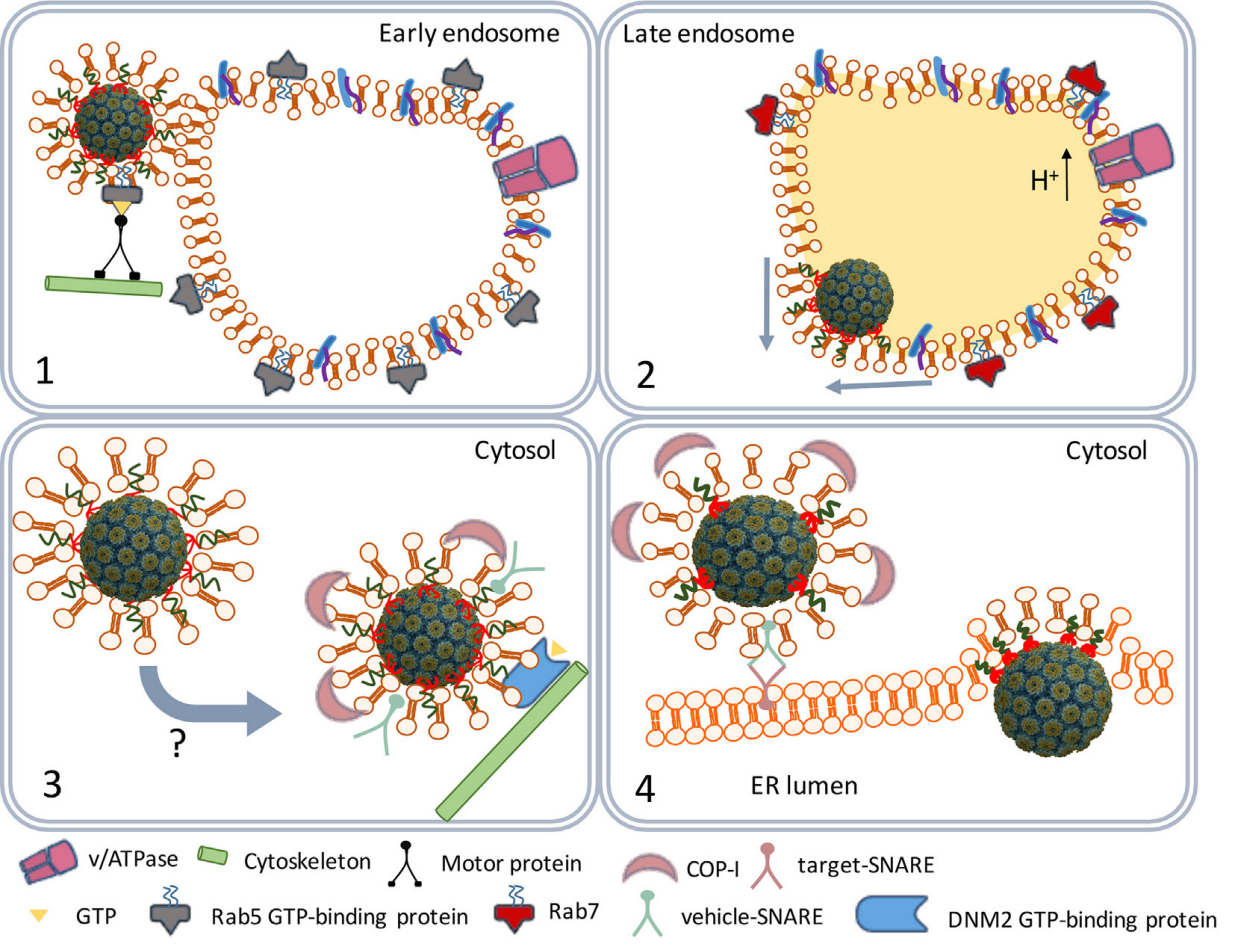
SV40 endocytosis and transport to the endoplasmic reticulum (ER). Scheme depicting the traffic of SV40-containing caveolar vesicles to early endosomes (1). The maturation of endosomes and fusion with lysosomes to endolysosomes (2). The traffic of coat protein I (COPI)-decorated vesicles to the ER (3). ER internalization (4).

The caveolar tyrosine kinases promote the recruitment of the cytoskeleton proteins actin and dynamin II (30, 34, 35) and assisted by Rab5 (a GTP-binding protein) and Arf1 (a GTP-ase) the caveolae traffic along the cytoskeleton to early endosomes (Figure 3) (36, 37). The SV40 particles remain bound to the membrane-associated GM1 molecules in endosomes during their maturation to late endosomes and endolysosomes. At this stage, the cell entry process of polyomaviruses differs from that of other viruses. Most viruses directly move from the endolysosome to the nucleus (38, 39). However, before they traffic to the nucleus, major part of their structural proteins is degraded by the lysosomal proteases yielding viral peptides which are loaded as antigens on MHC class II (MHC-II) molecules (40). MHC-II molecules are expressed in antigen-presenting cells (APCs) that are involved in the induction of adaptive immune responses.

Polyomaviruses, on the contrary, traffic from the endolysosome to the endoplasmic reticulum (ER) (36). The acidic environment in the endolysosome renders SV40 particles susceptible to successive disassembly steps later in the ER. However, before the virus particles are degraded by lysosomal enzymes they leave the endolysosome. The GM1 molecules that remained bound to the SV40 particles mediate the budding from the endolysosome membranes, it has remained unknown which factors are responsible for the timing of the budding process (41). The early exit from the endolysosomes prevents degradation of the SV40 structural proteins by lysosomal enzymes. As a result, SV40 antigens are not loaded on MHC-II molecules and presented by APCs to lymphocytes.

## ER Processing and Nuclear Entry

The virus-containing vesicles traffic from the endolysosome to the ER using the trans-Golgi network, a bidirectional vesicle trafficking route between ER and Golgi apparatus (Figure 3) (42). Trans-Golgi network vesicles are coated with coat protein I (COPI) complex proteins originating from the lysosomal membranes (43, 44). The COPI-coated vesicles containing the SV40 particles fuse with the ER membranes (45) releasing the virus particles into the ER lumen.

**Figure 4:**
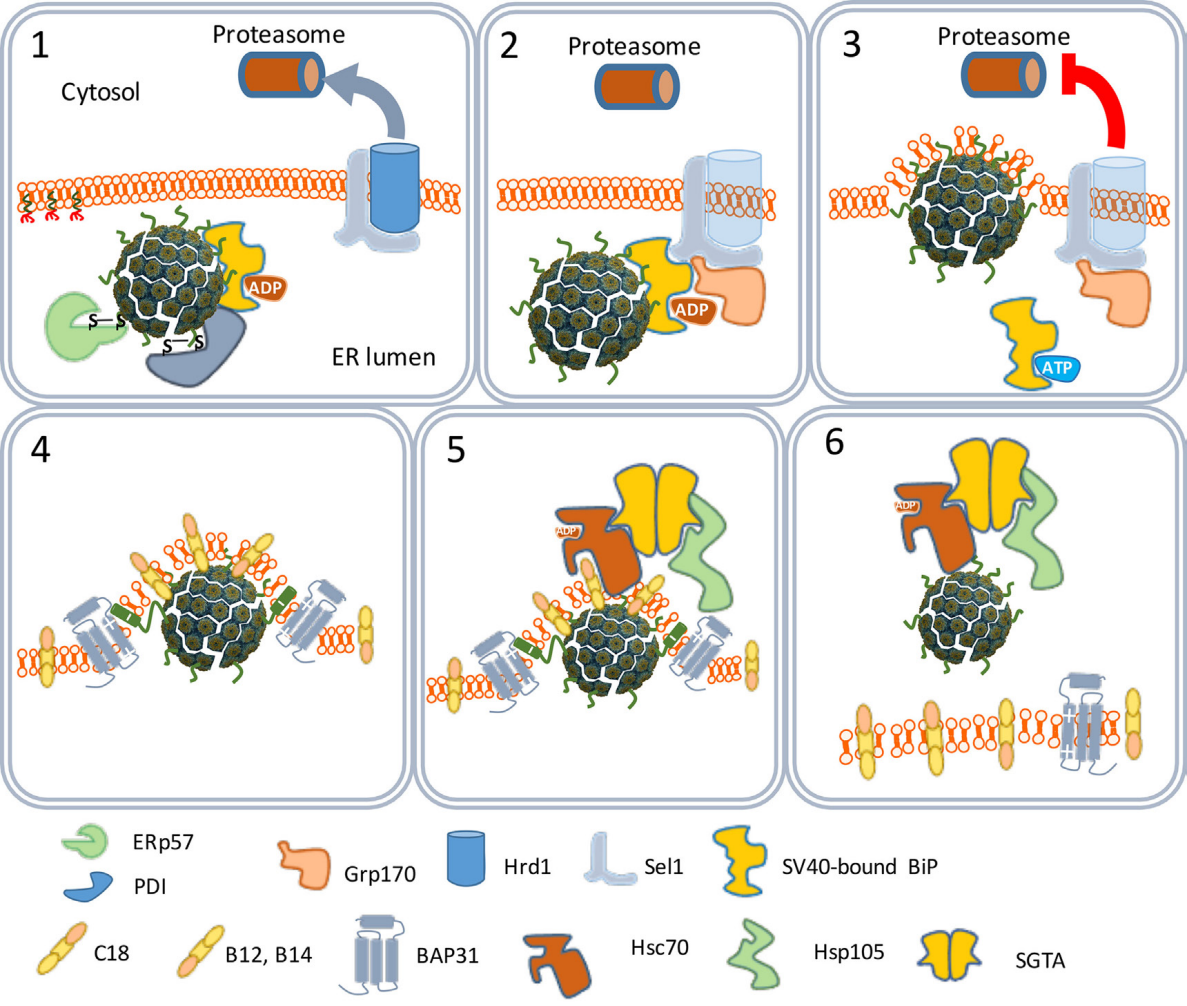
The traffic of SV40 particles from the endoplasmic reticulum (ER) to the cytosol. SV40 particles are destabilized by the ER-associated degradation machinery (1) and remain associated with the ER membrane (2). But instead of leaving the ER by binding Hrd1 and to be loaded to cytoplasmic proteasomes (3), the destabilized particles interact with ER membrane-resident proteins (4) and associate with cytosolic chaperones (5) to move to the cytosol (6).

The ER-associated degradation (ERAD) system is a protein quality control mechanism that recognizes nascent polypeptides and assists them in their correct folding or degradation by cytoplasmic proteasomes (46). The SV40 particles are recognized by the ERAD system as misfolded proteins. Peptide disulfide isomerase and ER protein 57 bind to and reduce the disulfide bonds that stabilize the VP1 pentamers (Figure 4). The pentamers become less tightly associated with each other and the VP2 and VP3 become exposed to the exterior (47). Indeed, *in vitro* studies confirmed that the SV40 particles in the ER are larger than those in the cytosol (48). The hydrophobicity of VP2 and VP3 renders the virus particles prone to aggregation. Aggregation is prevented by binding to the molecular chaperone BiP. Usually, proteins to be degraded bind a membrane-bound protein complex containing Hrd1 that targets them for degradation by cytoplasmic proteasomes. In this degradation process, specific peptides derived from the proteins are loaded as antigens on MHC-I molecules to be presented at the cell surface to cells of the host’s immune system. MHC-I molecules are involved in the induction of cellular immune responses. Polyomaviruses, however, do not bind Hrd1-containing complexes and are not loaded to proteasomes. SV40 thus has developed an effective mechanism to prevent being targeted for proteasome degradation (47, 49). This implies that SV40 is also capable of avoiding presentation on MHC-I molecules, thereby preventing the induction of cellular antiviral immune responses upon infection.

**Figure 5:**
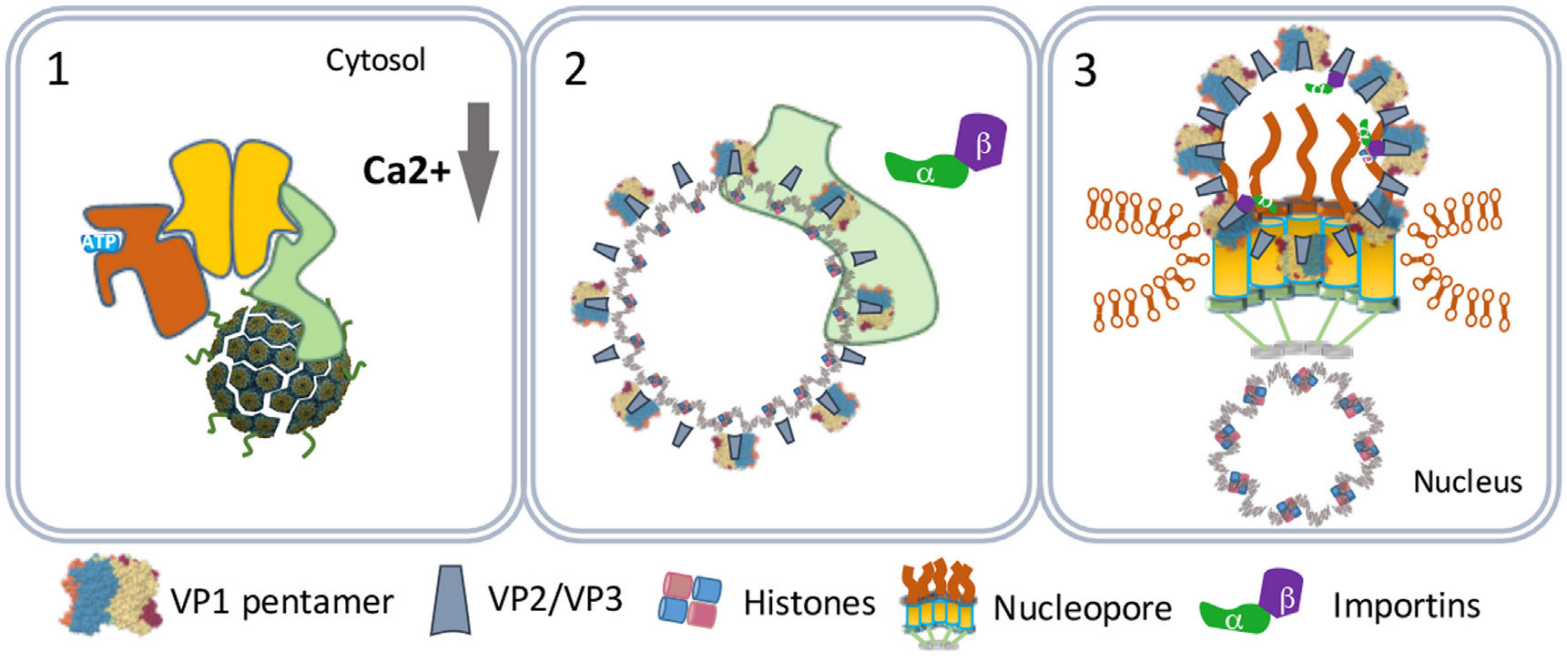
SV40 nuclear entry. SV40 particles further destabilize by low calcium in the cytosol (1) and lose VP1 by cytosolic chaperones (2). The particles bind nucleopores and viral genomes enter the nucleus (3).

One scenario to explain this phenomenon is that the virus particles use an extra step *via* the cytoplasm to evade proteasome degradation and reach the nucleus. In this scenario, the exit of the virus–BiP complexes from the ER to the cytosol is facilitated by proteins of the ERAD system in combination with cytosolic chaperones and takes place at particular domains on the ER membrane named foci (Figure 4) (50–52). At the foci, the destabilized virus particles are pulled-out from the ER and released into the cytoplasm (53). In the cytoplasm, VP1 is removed from the SV40 particles due to the action of chaperones (53) and the local physiological conditions (54). The nuclear localization signals present on VP2 and VP3 bind α/β importins (55–58) that mediate the transport of the SV40 genetic material into the nucleus through the nucleopores (Figure 5) (59–61).

**Figure 6:**
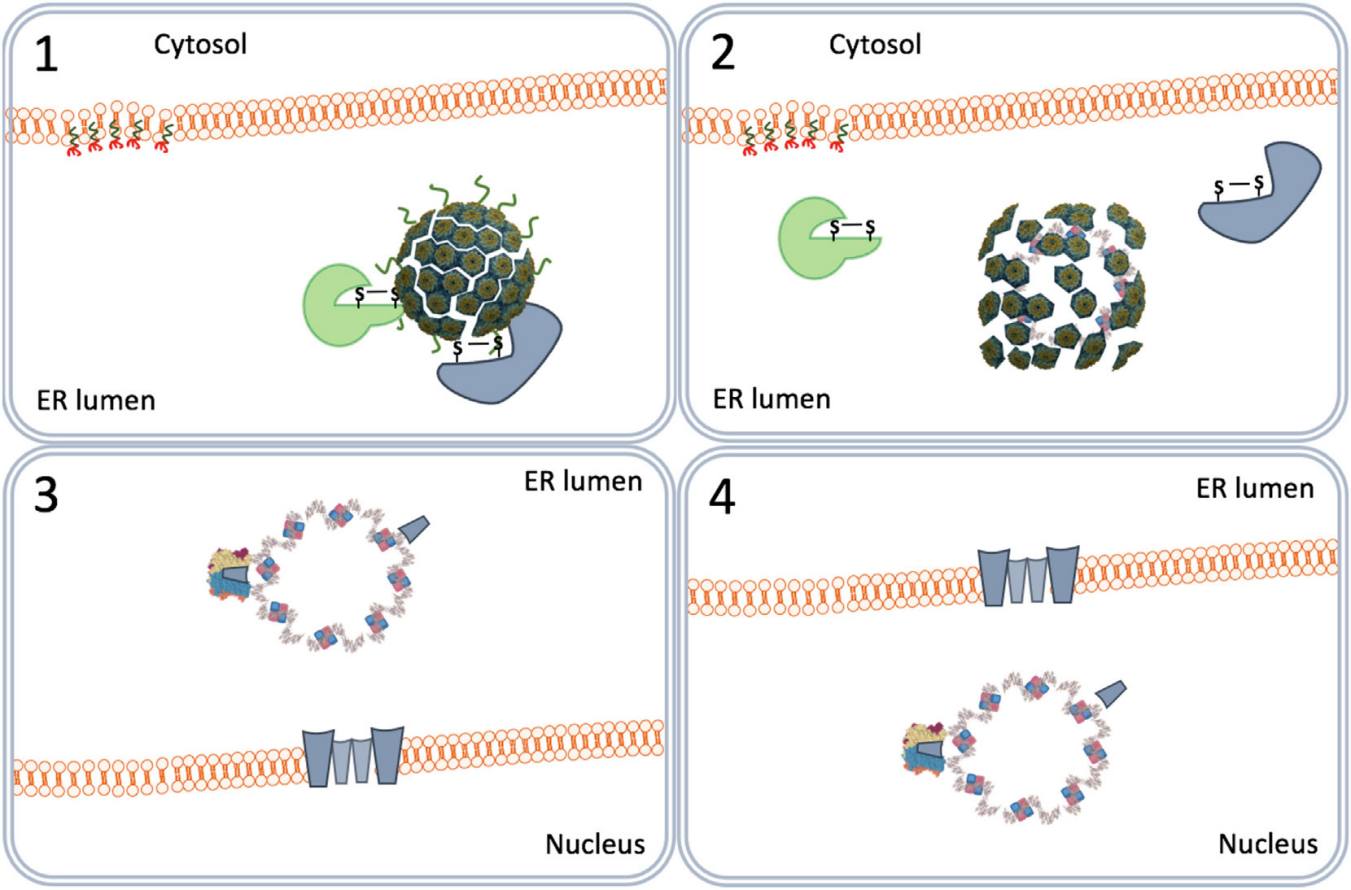
Nuclear entry by viroporins. The virus particles destabilized by the endoplasmic reticulum-associated degradation system (1) form VP2 and VP3 viroporins (2) on the inner nuclear membrane (3) that pull the viral genomes into the nucleus (4).

In another scenario, the virus particles directly move from the ER lumen to the nucleus. This scenario relies on the capacity of purified VP2 and VP3 monomers to insert in membranes forming pore-like structures named viroporins (62, 63). The ERAD-mediated destabilized SV40 particles allow the formation of VP2/VP3 viroporins on the inner nuclear membranes. The viroporins subsequently pull the SV40 genomes into the nucleus (Figure 6) (64).

## The Presence of SV40 in the Human Population

Polyomaviruses cause chronic symptomless infections in their hosts and since they are replication-competent sustained adaptive immune responses to the virus are induced in infected hosts (65–69). In humans, polyomaviral particles can be found in the kidney and urine of immunocompetent individuals and in kidney, brain, lung, or peripheral blood mononuclear cells in immunocompromised individuals.

Since the massive administration of children with SV40 in the fifties and sixties of the previous century, many studies have been performed to determine the consequences of this undesired vaccination. However, the epidemiological studies to identify SV40 seropositive individuals were inconclusive. The assays used in macaques to detect SV40 are not reliable in humans (70, 71). Only a small number of individuals vaccinated with SV40-contaminated polio vaccine developed antibodies to SV40 (16, 72) and the very low antibody titers suggest that SV40 does not replicate in human cells (73, 74). Finally, the presumed SV40 antibodies disappeared with time, indicating that the seropositive individuals were not chronically infected with SV40 (71, 75, 76). From a study with zoo workers that were in close contact with macaques for a long time, it was concluded that SV40 does not replicate in humans (70, 71, 74, 77). Overall, from all epidemiologic studies, the Institute of Medicine from the National Institutes of Health in the USA concluded that humans are not a host of SV40 and that SV40 is not a human pathogen (78).

## Concluding Remarks

The successful entry into a host cell is a crucial step in the virus replication cycle. Among all viruses, polyomaviruses including SV40 have developed a unique way of entering a permissive cell and expressing its genetic information in the nucleus of an infected cell. The viral particles prevent activation of TLRs, escape from the proteasome, and thus evade antigen presentation to cells of the host’s immune system during this initial stage of infection. The serological analysis of hosts naturally infected with polyomaviruses shows long-lasting adaptive immune responses, indicating that replicating polyomaviruses activate RLRs (79).

Epidemiological studies revealed that humans are not a host for SV40 and that this macaque polyomavirus does not replicate in humans. Therefore, the human population is considered to be immunologically naïve for SV40. On the basis of these findings, it is expected that replication-defective SV40 gene delivery vectors are completely non-immunogenic in humans.

In a number of reports, it has been shown that replication-defective viral gene delivery vectors such as vectors derived from AAV and HIV-1 induce immune tolerance to the transgene proteins when administered to hosts that are naïve to the cognate virus (80–83). These studies indicate that replication-defective SV40 vectors are ideally suited for inducing immune tolerance to the transgene proteins in humans (17, 18). This is crucial for designing effective gene replacement therapies where long-term transgene expression in the target tissue is required to cure patients from inherited diseases. In addition, the capacity of SV40 vectors to induce immune tolerance opens the way to treat autoimmune diseases by restoring the immune tolerance to primary self-antigens involved in the autoimmune tissue destruction.Restoration of immune tolerance to self-antigens using viral gene delivery vectors is named reverse viral vector vaccination and has been a longstanding goal in autoimmunity research. To date, diabetes mellitus type 1, multiple sclerosis, and arthritis are the best studied autoimmune diseases. In rodent models of these diseases, it has been shown that replication-defective AAV or HIV-1 vectors encoding the primary self-antigens of the disease highly efficiently protect and cure the treated animals from the autoimmune disease (84–89). With our rapidly increasing knowledge on immunology, the list of autoimmune diseases is growing and includes the major degenerative diseases of our time such as cardiovascular diseases (90), neurodegenerative and psychiatric diseases (91), obesity, diabetes mellitus type 2 (92, 93), arthritis and pulmonary diseases (94). Moreover, the induction of immune tolerance in recipients to MHC-I molecules of donor cells will improve the success rate of tissue transplantations.

HIV-1 derived vector particles are instable, rapidly degraded when administered *in vivo*, and for these reasons only used for *ex vivo* gene therapy to treat blood-related genetic disorders or cancer. To date, AAV vectors are the most popular for use in *in vivo* gene therapy. However, the majority of the human population encountered wild-type AAV together with its helper virus (adenovirus, causing the common cold) and developed an immune memory against the AAV capsid proteins. Clinical studies revealed that because of the immune memory in humans, the *in vivo* efficacy of AAV vectors is very low. SV40 vectors are the only gene delivery vectors that can be used for inducing immune tolerance to transgene proteins in humans and for this reason the oldest viral gene delivery vector will be key to the successful development of effective interventions for today’s major diseases (95).

## Author Contributions

MGT and PH wrote the manuscript and MGT designed the figures.

## Conflict of Interest Statement

Both authors are employed by Amarna Therapeutics. The company holds patents on the production and use of polyomaviral gene delivery vectors.
